# Central statistical monitoring in clinical trial management: A scoping review

**DOI:** 10.1177/17407745241304059

**Published:** 2025-01-02

**Authors:** Maciej Fronc, Michał Jakubczyk, Sharon B Love, Susan Talbot, Timothy Rolfe

**Affiliations:** 1 Decision Analysis and Support Unit, Institute of Econometrics, SGH Warsaw School of Economics, Warsaw, Poland; 2 Central Monitoring and Data Analytics, GSK, Warsaw, Poland; 3 MRC Clinical Trials Unit at UCL, University College London, London, UK; 4 Biostatistics, Amgen, Thousand Oaks, CA, USA; 5 Central Monitoring and Data Analytics, GSK, Brentford, UK

**Keywords:** Clinical trials, central statistical monitoring, central monitoring, risk-based monitoring, risk-based quality management, quality by design, data-driven approach

## Abstract

**Background:**

Clinical trials handle a huge amount of data which can be used during the trial to improve the ongoing study conduct. It is suggested by regulators to implement the remote approach to evaluate clinical trials by analysing collected data. Central statistical monitoring helps to achieve that by employing quantitative methods, the results of which are a basis for decision-making on quality issues.

**Methods:**

This article presents a scoping review which is based on a systematic and iterative approach to identify and synthesise literature on central statistical monitoring methodology. In particular, we investigated the decision-making processes (with emphasis on quality issues) of central statistical monitoring methodology and its place in the clinical trial workflow. We reviewed papers published over the last 10 years in two databases (Scopus and Web of Science) with a focus on data mining algorithms of central statistical monitoring and its benefit to the quality of trials.

**Results:**

As a result, 24 scientific papers were selected for this review, and they consider central statistical monitoring at two levels. First, the perspective of the central statistical monitoring process and its location in the study conduct in terms of quality issues. Second, central statistical monitoring methods categorised into practices applied in the industry, and innovative methods in development. The established methods are discussed through the prism of categories of their usage. In turn, the innovations refer to either research on new methods or extensions to existing ones.

**Discussion:**

Our review suggests directions for further research into central statistical monitoring methodology – including increased application of multivariate analysis and using advanced distance metrics – and guidance on how central statistical monitoring operates in response to regulators’ requirements.

## Background

According to the US Food and Drug Administration (FDA), centralised monitoring is defined as ‘a remote evaluation carried out by sponsor personnel or representatives (e.g. clinical monitors, data management personnel or statisticians) at a location other than the sites at which the clinical investigation is being conducted’.^1^ The European Medicine Agency (EMA) claims there is a potential to improve central monitoring to central statistical monitoring using statistical methodology to monitor the quality of the trial conduct and data.^2^ Central statistical monitoring is the most efficient way to ensure patient safety, trial integrity and data quality in multicentre clinical trials,^3–5^ and its use is recommended by both FDA and EMA, and in the ICH-E6(R2) Good Clinical Practice guidelines.^1,2,6^ Early implementation of central monitoring models focussed primarily on key risk indicators as a simple, implementable solution for identifying site-to-site variation.^7,8^ However, a more holistic approach to the data quality assessment via using complex statistical analytics enriches simple univariate assessment of key risk indicators and provides data-driven solutions for the detection of systemic issues, data irregularities and potential fraud. Information obtained from central statistical monitoring analyses is a roadmap of decision-making processes.^9,10^

Although it might seem reasonable to assume that the incidence of misconduct or fraud in science in general and in clinical trials, in particular, is low; the true incidence is difficult to estimate.^5^ This uncertainty recently led Smith to conclude that the problem of fraud in medical research ‘is huge’.^11^ This uncertainty highlights the need for additional research in the area of trial quality, with a specific focus on fraud detection methodology.

Kirkwood et al.^12^ brought together the thinking on central statistical monitoring and published R-programmes for others to use. They classified individual participant-level monitoring and site-level monitoring that was required and gave methods to look for recording and entry errors, procedural errors and fraud. They envisioned a time in the future when these checks would be automated and routinely carried out. Almost a decade later, trial sponsors have interpreted which checks to run and how and when to run them, and many have extended the methodologies. However, limited information is available on the methods used and the results of any checks are not routinely reported.

This review aims to answer the question of how central statistical monitoring is conducted in the process of the clinical trials with an emphasis on quality issues, and what kind of quantitative methods are applied already or are being developed. This approach provides practical knowledge about central statistical monitoring in terms of technical solutions and how it is used.

To answer these questions, the review of central statistical monitoring literature was conducted according to the approach on a scoping review recommended by Arksey and O’Malley and supported by the PRISMA guideline by mapping key concepts underpinning the chosen research area settled in a broader theoretical framework.^13–15^ The chosen approach helps to identify in central statistical monitoring research gaps and directions, and investigate current practices and also their barriers.

## Methods

### Literature search and method extraction

Two large citation electronic databases were searched – Scopus and Web of Science. In the search, we used the following keywords which define the research axis of the review (Table 1). The query was developed in agreement between all authors of this article.

The search was conducted on papers published in English between January 2012 and April 2022 to give a 10-year span. We excluded from the search books and book chapters as secondary literature sources. The papers identified in the search were subsequently analysed, and we included or excluded papers which met any of inclusion/exclusion criteria. All of the criteria are provided in Supplemental Material 1.

### Categorisation of the papers

The selected papers provide information about currently used and developed quantitative methods, and deal with the topic of quality issues in the use of central statistical monitoring. The selected papers were categorised in terms of their content into three categories: (a) ‘Central statistical monitoring processes’ focuses on central statistical monitoring in terms of quality issues and clinical trial workflow; (b) ‘Established central statistical monitoring methods’ refers to methods currently used within central statistical monitoring in different configurations; (c) ‘Innovative central statistical monitoring methods’ deals with techniques being developed for improving the central statistical monitoring methodology. In this context, innovation refers to techniques which extends beyond the basic methodology widely used in central statistical monitor’s daily practice.

## Results

**Figure 1:**
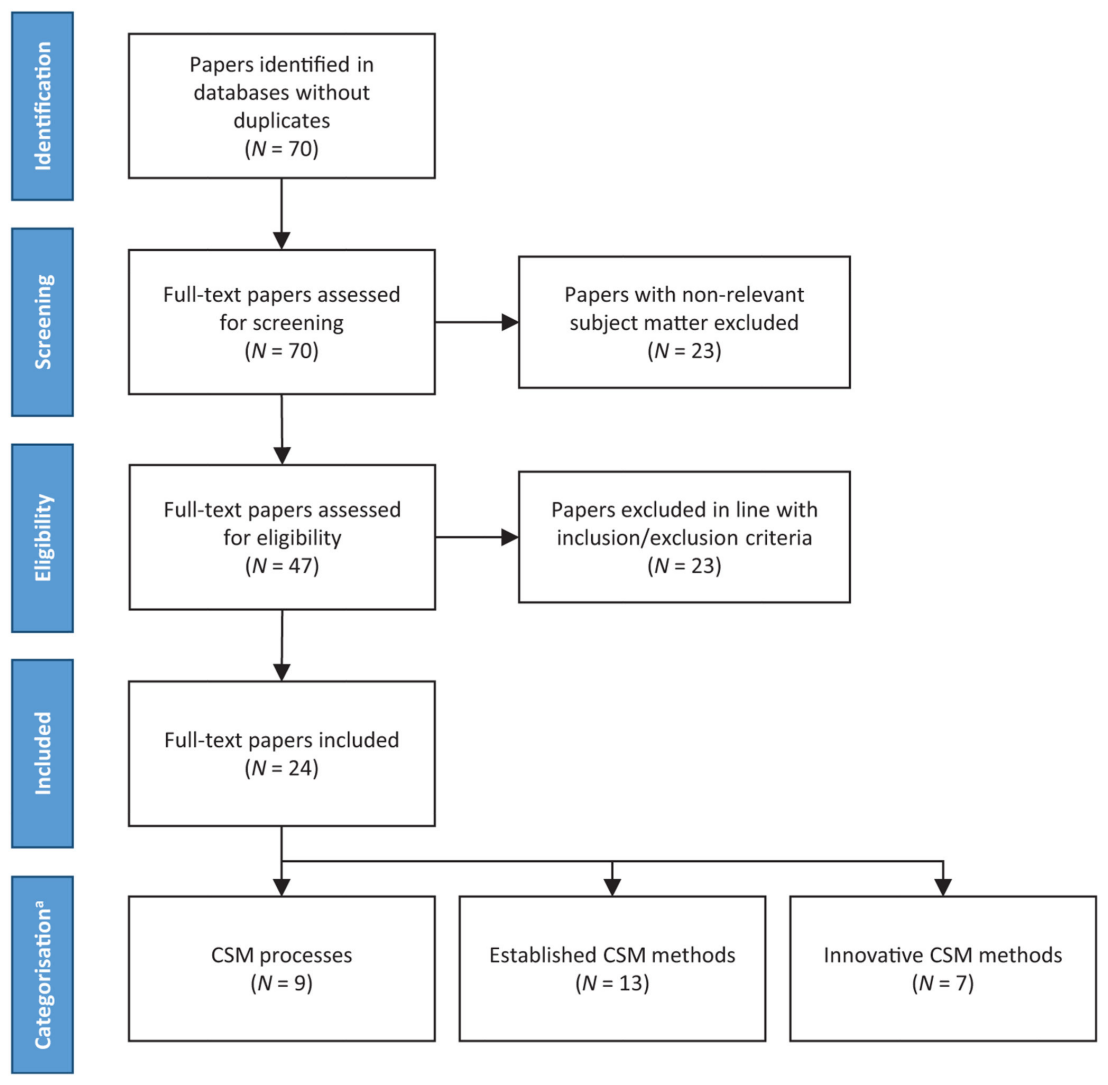
Stages of search process for the review. ^a^The total number of papers from this category is greater than the total number of included articles because a part of them was classified into more than one category.

The title and abstract review yielded initially 70 papers according to the applied query, with 24 included in this article as they met the predefined selection criteria. All of them were split into the predefined categories and it was possible for a paper to be classified into more than one category. Figure 1 depicts the whole literature selection process; and Table 2 shows included articles classified to defined categories.

### Central statistical monitoring processes

Kirkwood et al.^12^ characterised central statistical monitoring as a more cost-effective alternative than full onsite monitoring, where data checks are performed by the coordinating centre, avoiding the need to visit sites as frequently. Both approaches can coexist in a proportion sufficient to improve the quality and reduce costs. In turn, Venet et al.^16^ described central statistical monitoring as a tool to detect abnormal patterns among clinical data and to help focus on sites which actually need to be monitored in detail. Central statistical monitoring aims to avoid quality issues in the study conduct and the final outcome as well. In this context, quality can be defined as a set of product or process characteristics which arises from protecting patient’s well-being and providing a reliable assessment of investigated treatment.^9^ Buyse et al.^17^ perceive central statistical monitoring as an improvement of quality management which relies on statistical indicators estimated on the basis of data collected across the whole trial, in turn Pogue et al.^18^ consider central statistical monitoring more precisely as a part of quality assurance.

Central statistical monitoring can be considered in different ways – from assuring data integrity to data analytics which helps to leverage information hidden in clinical data. This scope translates into the quality of the study outcome, which should be handled at all levels of the trial. As the expansion of clinical trials is progressing and requires more resources to be engaged, it is recommended to take measures respectively to the risk level. The paradigm of central statistical monitoring (and the entire risk-based approach as well) needs to be incorporated into the clinical trial workflow to ensure the synergy with current processes. The extension of this thought is available in Supplemental Material 2.

### Established central statistical monitoring methods

The earliest central statistical monitoring methods typically relied on simple univariate statistical methods. These were used either individually or collectively to provide a more comprehensive understanding of the study conduct. Oba among others presented univariate approach within unsupervised learning to detect atypical data patterns.^19^ He compared variable distribution between the sites and summarised them by descriptive statistics, such as mean, median, standard deviation, skewness or kurtosis. The distribution analysis was completed by data visualisation which is complementary to numeric results and facilitates their interpretation. Knepper et al.^20^ adopted a similar approach, investigating the study in terms of non-random error detection supported with data visualisation to make received statistics more understandable.

Knepper et al.^20^ also conducted correlation check to examine the relationships between variables and recognise unusual data patterns. The method compares variables pairwise within a certain site. Kirkwood et al.^12^ compared the correlation structure between sites aiming to identify outlying ones. In turn, Pogue et al. investigated the correlation of repeated measurements expressed by an intraclass correlation coefficient. This approach helps to find out fabricated data by flagging cases with high similarities of continuous measurements recorded multiple times.^18^

A battery of standard statistical tests is usually applied for comparing patients between sites.^21^ For example, Afroz et al. performed one-way analysis of variance (ANOVA) to compare two or more samples aided with post hoc analysis that helps to distinguish groups different from others. Another test they used was the chi-square goodness-of-fit test which evaluates two or more samples in terms of similarity as well.^22^ Kirkwood et al.^12^ recommend variance check as it helps to monitor sites in terms of repeated measurements. Lower variance may indicate fabricated data at the site or patient level. Statistical tests are recommended for categorical data as well. Chi-square test of independence is applied to investigate patients’ responses distribution among predefined categories. Kirkwood et al.^12^ suggest to use the test in the case of evaluation of safety, treatment compliance, and the primary efficacy endpoints. In turn, Buyse et al.^17^ suggest to apply many tests rather than only a few of them and call it the essence of central statistical monitoring. This approach is more efficient than single tests because it discloses knowledge hidden behind evidence accumulated across the entire study. Overall statistical testing makes better use of potential included in clinical data than focusing only on single metrics.

Descriptive statistics (frequency analysis) and statistical testing are used for digit preference analysis. According to the work by Knepper et al.,^20^ digit preference can be investigated by comparing digit frequency distribution of a single subject to other ones using chisquare or Fisher’s exact test. Alternatively, the comparison can be performed using mean and standard deviation in relation to a confidence interval. Another approach is based on the fact that every numeric result is considered as a sequence of digits. In the case of numbers with leading digits from 1 to 9, frequency of others should follow Benford’s distribution. If clinical data are inconsistent with the distribution, it implies a fraudulent activity.^12,22^ Oba proposed another approach to examine digit preferences in a particular measurement, that is, volcano plot.^19^ It is a scatter plot where *y*-axis represents a negative common logarithm of a raw *p*-value from testing digit preference, and the *x*-axis is a maximum difference in the frequency distribution of all digits between a single site and all other sites. Then, the most outlying upper points on the plot indicate suspected sites (Supplemental Material 3, Figure S3–1).

Multiple statistical testing has another meaning for testing data consistency. Trotta et al.^23^ noticed that the abundance of statistical tests gives too many results to analyse them one by one; therefore, data aggregation is needed to facilitate the analysis. All performed tests can be transformed into one metric known as a data inconsistency score which is a weighted geometric mean of the *p*-values of all tests comparing site *i* with all other sites, and can be calculated by the following formula:

(1) $$DIS_{i}=\exp\left(\frac{1}{\Sigma_{j=1}^{N}w_{j}}\overset{N}{\underset{j=1}{\sum }}w_{j}\log p_{ij}\right)$$

where *DIS*_*i*_ is the data inconsistency score for site *i, w*_*j*_ is a weight proportional to the correlation between the tests, and *p*_*ij*_ is a *p*-value of *j*th test in site *i*. This operation transforms the outcome of all performed statistical tests into a one metric which makes sites easily comparable to each other, and helps to identify outliers considering the value obtained.^17^ Actually, calculating the data inconsistency score is more complicated, but the simplified definition is sufficient for these considerations.^23^

The topic of dimensionality reduction is substantial for clinical data analysis, as clinical trials handle many variables within a single study. These kinds of methods analyse leveraging the information load form more than one variable. It is more useful in the context of inliers and outliers detection than univariate approach, ensuring more comprehensive analysis.^19^ Pogue et al.^18^ consider multivariate methods in a greater extent, that is, from statistical summaries through statistical tests to purely graphical methods. Multivariate analysis closely relies on distance metrics which determine proximity (similarity) of data points. A particularly important metric within this category is the usage of Mahalanobis distance which Afroz et al.^22^ found as a multidimensional risk assessment method. Mahalanobis distance works similarly to Euclidean distance, but raw data are normalised by the variance, which takes into account the correlations between variables. Olsen et al.^24^ recognised Mahalanobis distance as the best suited method for multivariate pattern and anomaly detection. Venet et al.^16^ used principal component analysis to reduce dimensionality and presented results in the two-dimensional space. Two dimensions are desired to facilitate visualisation of results in terms of outlier detection and make results more understandable.

The most comprehensible and attractive form of presenting study outcome is data visualisation. It is a ubiquitous method within cited aforementioned papers. Data visualisation usually completes performed analyses, which makes statistical concepts and raw data more tangible for a viewer via a graphical representation of the raw or aggregated data. Processes can be visualised as well, which helps to monitor them through the time. Van den Bor et al.^25^ presented the process of patient enrolment as a cumulative function and summarised with a boxplot to detect suspected sites. Afroz et al.^22^ compared sites using boxplots considering Mahalanobis distance between them. Buyse et al.^26^ used boxplots to compare *p*-values of the Cochran– Mantel–Haenszel test to the proportion of patient with errors. Edwards et al.^27^ monitored a trial using density plots which showed a distribution of the coefficient of variation for continuous variables (Supplemental Material 3, Figure S3–2).

Cited researchers have already evaluated the effectiveness of various methods based on the type of clinical data they used. The last two columns in Table 3 show differences between implemented datasets. Some of them include cases labelled as fraud, which enables to evaluate the performance of tested techniques using metrics, such as sensitivity, specificity or detection rate.^12,18,20,21,23,25^ However, Buyse et al. used labelled data differently. The initial data set was enriched with simulated erroneous data to examine the impact of their kind (random or systematic) on the study outcome.^26^ However, analyses performed on unlabelled data just focused on flagging suspicious data points needed to be verified individually.^16,17,19,22,24,27^ This approach needed the expert knowledge or experience of the study team to build the medical context which helps to identify the potential cause.

### Innovative central statistical monitoring methods

While the established central statistical monitoring methods perform satisfactorily, there is room for further improvement. The elusive nature of data errors and fraud, due to their subtlety, presents a challenge. Therefore, there is a need for expanding the range of tools further to make error and fraud detection more robust (Table 4).

Churovaét al.^28^ conducted anomaly detection using clustering with seven distance metrics and their configurations. The applied metrics are as follows: Canberra, Chebyshev, Cosine, Euclidean, Manhattan, Mahalanobis and Minkowski. A distance matrix was determined per single metric and their derivatives as well. Calculated distances were examined using univariate testing. Clustering was performed using thresholds calculated individually for each metric twice: with predefined percentiles and following the interquartile range rule. As a result, a combination of Canberra, Manhattan and Mahalanobis gave the best performance, whereas Cosine and Chebyshev underperformed and were excluded from further analysis. Ultimately, Churova et al. achieved the optimal number of patients to investigate in the range 5.0%–22.5% of total number of patients.

Zink et al.^29^ addressed the development of the multivariate analysis methodology for inliers and outliers identification, and how single variables contribute to each multivariate outlier. They proposed squared Mahalanobis distance which is proportional to Hotelling’s *T*-squared distribution. The distribution is dedicated to handling multivariate hypothesis testing. On the basis of the distribution, a confidence interval was constructed as a criterion for outlier identification. This approach can be supported with a scatterplot as a control chart for monitoring suspected values. The authors summarised findings by visualising the proportion of the squared Mahalanobis distance on a pareto plot (Supplemental Material 3, Figure S3–3). This helps to investigate outliers in terms of the most contributing variables, and hence points out areas for risk mitigation.

Hatayama and Yasui suggest Bayesian finite mixture model to detect atypical patterns across small and moderate studies.^30^ The methods aims to estimate a mixture distribution which takes into account sub-populations in an overall dataset – that is, fraudulent and non-fraudulent cases. Each of these categories has its own distribution, whereby the fraudulent one is a minority. Bayesian interference is used to estimate parameters of the models, but only the majority distribution is used by comparing averaged site results with it.

Desmet et al.^31^ proposed an application of the beta-binomial distribution for modelling event counts. They used it for estimating a hybrid model which involves fraudulent and non-fraudulent sub-populations. The detection procedure relies on the following steps: (a) fit a beta-binomial model to the overall data and (b) assign a *p*-value to each site and classify respectively to a significance level (*α*_cr_). This criterion indicates which cases are fraudulent or not.

Desmet et al.^32^ proposed as well an application of the linear mixed-effects model to evaluate differences between single sites and the entire study. They modelled a heterogeneous population composed of ‘normal’ and ‘abnormal’ observations. Each of those groups follows separate distributions with shifted means. The authors assumed a simple detection algorithm as a baseline for the model they investigated. According to the assumption, first, a hybrid model is estimated (including all of the data). A *p*-value is assigned to each observation. Then, observations are flagged respectively to assumed *α*_cr_. However, Desmet et al. noted that the model is not always adequate for hybrid data and underperforms in terms of discrepancy detection. Therefore, they applied linear mixture-effects models to detect atypical sites across a study, which are dedicated to clustered data. *P*-values are assigned to each observation respective to the normal distribution estimated on the basis of the average variance calculated between a single site and the rest of them. Then, sites are flagged in relation to *α*_cr_.

Pogue et al.^18^ proposed logistic regression for fraud prediction by establishing cut-offs for the risk-scoring process. The authors selected predictors and eliminated redundancy within them using principal component analysis. They compared five three-variable models that have same two predictors. The predictors are associated with a risk level as a result of a statistical test performed for chosen attributes of the study. The risk was considered on a five-level scale in the range of 0–4. On this basis, the logistic model was estimated to predict the total risk level as a sum of scores assigned to predictors. Each model has its own cut-offs since it differs from the other with the third predictor. All models can be analysed at different risk levels and summarised in terms of a number of fraudulent cases, but it also indicates which risk level the dubious cases start from.

Zink et al.^33^ proposed funnel plots as a traffic-light system which indicates risk in terms of the duration of patient follow-up. The authors analysed discontinuation rate versus total number of patients. This relationship is presented on a scatterplot with constant thresholds that indicate severity of the risk. This graph works similarly as a control chart to monitor a process (Supplemental Material 3, Figure S3–4). The authors moved away from an assumption that the risk is independent of the number of patients to apply asymptotic thresholds. The higher risk is observed for smaller sites and it gradually decreases proportionate to increasing number of patients. Zink et al. proposed how to calculate the thresholds based on the asymptotic confidence interval of the event rate. This method was presented through the prism of two perspectives: binary outcomes, where events are counted zero-one independently to their repetition, and count-type outcomes, where the number of event repetitions is taken into account. In the first case, the confidence interval is determined on the basis of the observed event rate at the site, and the second one uses the Poisson distribution. Funnel plots turned out to be a useful graphical technique that considers changes in a sample size, patients exposure, and risk monitored through the time.

## Discussion

The objectives of the scoping review were to define an established framework for the central statistical monitoring process in the context of the study conduct, and identify quantitative methods underlying this approach including innovations. The invention of the method of central statistical monitoring is a response to regulators’ requirements and guidelines which recommend the risk-based approach for the monitoring of clinical trials. Central statistical monitoring is in the process of continuous improvement at conceptual, strategic and operational levels. It results in the development of monitoring processes aided by data mining algorithms.

Researchers and practitioners perceive central statistical monitoring as a part of the quality management in clinical trials. This point of view is consistent with regulators’ recommendations as they require the implementation of a quality management system across the entire clinical trial workflow. The pivotal factor that contributes to the quality of the final study outcome is data consistency. Consistency of the data is considered equivalent to the data quality and can be measured using the wide range of quantitative methods aided with data visualisation.

The outcome of well-performed central statistical monitoring analyses is a roadmap for high-quality decision-making. The reliability of the results determines the rationality of decisions and the future of the clinical trials in the long terms. The data-driven approach is not a substitute of decision-making based on human perception but a support in making optimal decisions. Data-driven decision-making is consistent with the idea of risk-based approach which involves the risk-based quality management strategy with risk-based monitoring as its key component. This strategy aims to manage tasks proportional to the perceived risk level, which allows the organisation to avoid unnecessary resource exploitation. Then, it makes the study conduct more efficient, which creates value for stakeholders of the drug development process and rises its quality. As a result, the risk-based approach forms a new dimension of the quality management.

To implement a risk-based quality management strategy effectively, it is necessary to incorporate central statistical monitoring into the clinical trial workflow. Its place in the process results from the existence of issues occurring in the data generation processes that fail to meet those defined in the study protocol. Discrepancies identified in clinical data provide information which is a starting point for determining corrective actions. They can be caused by fraud or just simple mistakes committed during the course of the study, but its signals are hidden among the collected data. These cases tend to leave some clues which may be identified using data mining algorithms.

Central statistical monitoring can be carried out with a battery of established methods, which are well known to practitioners. These methods were grouped into categories, such as statistical testing, descriptive statistics, data inconsistency score evaluation, correlation analysis, digit preferences check, multivariate analysis, and data visualisation. These methods are applied in various configurations, as their outcomes complement each other. Their usage is recognised as a must-have within the framework of central statistical review. In our opinion, the biggest potential lies in multivariate techniques as clinical trials are analysed in the multidimensional space outlined by variables involved in the study. Associations between these variables are not obvious for a viewer at first glance. Therefore, multivariate methods seem to be more powerful^19^ compared with the univariate approach which might be insufficient.^24^

Multivariate analysis discloses hidden data patterns and trends resulting from the natural multicollinearity in clinical data.^18,29^ Data visualisation is a powerful field as well, being ubiquitous among cited articles, thus proving its utility. Graphical representation of the data provides easy-to-interpret summaries of performed statistical analyses. Graphics can be shared with other people, without the need for detailed statistical knowledge, to communicate analysis results in an accessible way.

Although some methods are already established, their performance is insufficient to extract all valuable information included in the collected data. The need for innovations in the central statistical monitoring methodology is highlighted in the literature.^12,17^ A part of the methods was tested in simulated data which do not fully reflect the real nature of clinical data. It is recommended to improve the current methodology by refining available algorithms on data from actual studies. Cited researchers addressed the development of methods using multivariate analysis, mixed-effect models, data visualisation, and so on. Of course, it does not exhaust directions of further research but confirms the aforementioned statement about method usability.

Our objective in this scoping review was to consider the central statistical monitoring methods currently available in the literature. The publications mainly show the improvement in the data through finding these errors. Proving the value of central statistical modelling compared to other approaches, for example, onsite monitoring, is outside the remit of this study but has been discussed in other sources.^8,12,16,34–36^ Moreover, this approach is recommended by both good clinical practice and regulatory agencies.^1,2,6^ This scoping review summarises the concept of central statistical monitoring and identified methods used in this field or being developed. The extracted knowledge was consolidated into one consistent image of central statistical monitoring focusing on quality issues. The method identification showed what is the current state of the central statistical monitoring methodology development and what directions are noteworthy for further research on data mining algorithms.

## Supplementary Material

Supplemental material for this article is available online.

Supplementary Material

## Figures and Tables

**Table 1:** Keywords used in the query.

Part of the query	Purpose (research axis)
(‘clinical trials’ AND (‘central* monitoring’ OR ‘statistical monitoring’))	Application field
‘quality’ OR ‘management’	Process and quality context
(‘machine learning’ OR ‘data mining’ OR ‘artificial intelligence’ OR statistic* OR econometric*)	Expected methodology
(fraud* OR manipulation OR fabrication OR misconduct OR ‘systematic error’)	Problem to be solved

**Table 2:** Total summary of included papers.

No.	Author	Year	DOI	CSM processes	Established CSM methods	Innovative CSM methods
1.	Afroz et al.	2021	10.1016/j.cct.2021.106368			+
2.	Churova et al.	2021	10.2196/27172		+	
3.	Cragg et al.	2021	10.1177/1740774520976561	+		
4.	de Viron et al.	2021	10.1007/s43441-021-00341-5			+
5.	Li et al.	2021	10.1097/AOG.0000000000004513	+		
6.	Olsen et al.	2021	10.1186/s12874-021-01344-4			+
7.	Buyse et al.	2020	10.1007/s10147-020-01726-6	+		+
8.	Hatayama and Yasui	2020	10.1016/j.conctc.2020.100566	+	+	
9.	Trotta et al.	2019	10.1177/1740774519862564			+
10.	Zink, Castro-Schilo et al.	2018	10.1002/pst.1903		+	
11.	Zink, Dmitrienko et al.	2018	10.1177/2168479017738981		+	
12.	Buyse et al.	2017	10.1177/1740774517716158			+
13.	Desmet et al.	2017	10.1080/19466315.2016.1164751		+	
14.	van der Bor	2017	10.1016/j.jclinepi.2017.03.018			+
15.	Herson	2016	10.1007/s10147-015-0876-6	+		
16.	Knepper et al.	2016	10.1177/2168479016630576			+
17.	Oba	2016	10.1007/s10147-015-0914-4			+
18.	Timmermans et al.	2016	10.1007/s10120-015-0533-9	+		
19.	Timmermans et al.	2016	10.1007/s10147-015-0877-5	+		
20.	Desmet et al.	2014	10.1002/sim.6294		+	
21.	Edwards et al.	2014	10.1177/1740774513514145			+
22.	Kirkwood et al.	2013	10.1177/1740774513494504	+		+
23.	Pogue et al.	2013	10.1177/1740774512469312		+	+
24.	Venet et al.	2012	10.1177/1740774512447898	+		+

**Table 3:** Summary of papers in terms of basic method usage.

No.	Author	Year	Statistical testing	Descriptive statistics	DIS	Correlation analysis	Digit preference	Multivariate analysis	Data visualisation	Clinical data	Fraudulent cases
1.	de Viron et al.	2022	+		+				+	Original	Known
2.	Afroz et al.	2021	+	+			+	+	+	Simulated	Unknown
3.	Olsen et al.	2021		+				+	+	Original	Unknown
4.	Buyse et al.	2020	+		+				+	Original	Unknown
5.	Trotta et al.	2019	+		+				+	Modified	Known
6.	Buyse et al.	2017	+						+	Modified	Known
7.	van den Bor et al.	2017				+	+		+	Original	Known
8.	Knepper et al.	2016		+		+	+	+	+	Modified	Known
9.	Oba	2016		+			+	+	+	NA	Unknown
10.	Edwards et al.	2014		+					+	Original	Unknown
11.	Kirkwood et al.	2013	+	+		+	+		+	Modified	Known
12.	Pogue et al.	2013	+	+		+	+	+	+	Original	Known
13.	Venet et al.	2012						+	+	Original	Unknown

Note: ‘ + ‘ means the method is present in the article; and ‘NA’ means the information is not available. Three categories of clinical data were distinguished: ‘original’ – from real clinical trials without any modifications; ‘modified’ – original clinical data partially modified and ‘simulated’ – fully modelled on original clinical data. In addition, two categories of fraudulent cases were defined: ‘known’ – fraudulent cases are pointed out in the dataset; and ‘unknown’ – data unlabelled in terms of fraudulent activity.

**Table 4:** Summary of papers in terms of methods in development.

No.	Authors	Year	Method
1.	Churová et al.	2021	Clustering with seven different distance metrics to identify a combination of them with the best anomaly detection
2.	Hatayama and Yasui	2020	Bayesian finite mixture model for atypical sites detection
3.	Zink, Castro-Schilo et al.	2018	Squared Mahalanobis distances for outlier identification and assessment of the individual variable’s contribution to multivariate outliers
4.	Zink, Dmitrienko et al.	2018	Funnel plots for monitoring discontinuation rate
5.	Desmet et al.	2017	Statistical modelling using beta-binomial distribution for detection of atypical sites in terms of probability of some events
6.	Desmet et al.	2014	Linear mixed-effects models for differences identification between sites
7.	Pogue et al.	2013	Logistic regression for risk prediction

## References

[1] Food and Drug Administration (FDA) (2013). Guidance for industry oversight of clinical investigations – a risk-based approach to monitoring guidance for industry oversight of clinical investigations.

[2] European Medicines Agency (EMA) (2013). Reflection paper on risk based quality management in clinical trials.

[3] Agrafiotis DK, Lobanov VS, Farnum MA (2018). Risk-based monitoring of clinical trials: an integrative approach. Clin Ther.

[4] Baigent C, Harrell FE, Buyse M (2008). Ensuring trial validity by data quality assurance and diversification of monitoring methods. Clin Trials.

[5] George SL, Buyse M (2015). Data fraud in clinical trials. Clin Investig (Lond).

[6] International Council for Harmonisation of Technical Requirements for Pharmaceuticals for Human Use (ICH) (2016). Integrated addendum to ICH E6(R1): guideline for good clinical practice E6(R2).

[7] TransCelerate Biopharma Inc (2012). RBM interactive guide.

[8] Wilson B, Provencher T, Gough J (2014). Defining a central monitoring capability: sharing the experience of TransCelerate BioPharma’s approach, Part 1. Ther Innov Regul Sci.

[9] Timmermans C, Venet D, Burzykowski T (2016). Data-driven risk identification in phase III clinical trials using central statistical monitoring. Int J Clin Oncol.

[10] Fronc M, Jakubczyk M (2022). From business to clinical trials: a systematic review of the literature on fraud detection methods to be used in central statistical monitoring. Przegląd Stat.

[11] Smith R (2022). Time to assume that health research is fraudulent until proven otherwise?.

[12] Kirkwood AA, Cox T, Hackshaw A (2013). Application of methods for central statistical monitoring in clinical trials. Clin Trials.

[13] Arksey H, O’Malley L (2005). Scoping studies: towards a methodological framework. Int J Soc Res Methodol Theory Pract.

[14] (2018). PRISMA for scoping reviews.

[15] Munn Z, Peters MDJ, Stern C (2018). Systematic review or scoping review? Guidance for authors when choosing between a systematic or scoping review approach. BMC Med Res Methodol.

[16] Venet D, Doffagne E, Burzykowski T (2012). A statistical approach to central monitoring of data quality in clinical trials. Clin Trials.

[17] Buyse M, Trotta L, Saad ED (2020). Central statistical monitoring of investigator-led clinical trials in oncology. Int J Clin Oncol.

[18] Pogue JM, Devereaux PJ, Thorlund K (2013). Central statistical monitoring: detecting fraud in clinical trials. Clin Trials.

[19] Oba K (2016). Statistical challenges for central monitoring in clinical trials: a review. Int J Clin Oncol.

[20] Knepper D, Lindblad AS, Sharma G (2016). Statistical monitoring in clinical trials: best practices for detecting data anomalies suggestive of fabrication or misconduct. Ther Innov Regul Sci.

[21] de Viron S, Trotta L, Schumacher H (2022). Detection of fraud in a clinical trial using unsupervised statistical monitoring. Ther Innov Regul Sci.

[22] Afroz MA, Schwarber G, Bhuiyan MAN (2021). Risk-based centralized data monitoring of clinical trials at the time of COVID-19 pandemic. Contemp Clin Trials.

[23] Trotta L, Kabeya Y, Buyse M (2019). Detection of atypical data in multicenter clinical trials using unsupervised statistical monitoring. Clin Trials.

[24] Olsen MH, Hansen ML, Safi S (2021). Central data monitoring in the multicentre randomised SafeBoosC-III trial – a pragmatic approach. BMC Med Res Methodol.

[25] van den Bor RM, Vaessen PWJ, Oosterman BJ (2017). A computationally simple central monitoring procedure, effectively applied to empirical trial data with known fraud. J Clin Epidemiol.

[26] Buyse M, Squifflet P, Coart E (2017). The impact of data errors on the outcome of randomized clinical trials. Clin Trials.

[27] Edwards P, Shakur H, Barnetson L (2014). Central and statistical data monitoring in the Clinical Randomisation of an Antifibrinolytic in Significant Haemorrhage (CRASH-2) trial. Clin Trials.

[28] Churová V, Vyškovský R, Maršárlsová K (2021). Anomaly detection algorithm for real-world data and evidence in clinical research: implementation, evaluation, and validation study. JMIR Med Informatics.

[29] Zink RC, Castro-Schilo L, Ding J (2018). Understanding the influence of individual variables contributing to multivariate outliers in assessments of data quality. Pharm Stat.

[30] Hatayama T, Yasui S (2020). Bayesian central statistical monitoring using finite mixture models in multicenter clinical trials. Contemp Clin Trials Commun.

[31] Desmet L, Venet D, Doffagne E (2017). Use of the beta-binomial model for central statistical monitoring of multicenter clinical trials. Stat Biopharm Res.

[32] Desmet L, Venet D, Doffagne E (2014). Linear mixed-effects models for central statistical monitoring of multicenter clinical trials. Stat Med.

[33] Zink RC, Dmitrienko A, Dmitrienko A (2018). Rethinking the clinically based thresholds of transcelerate biopharma for risk-based monitoring. Ther Innov Regul Sci.

[34] Adams A, Adelfio A, Barnes B (2023). Risk-based monitoring in clinical trials: 2021 update. Ther Innov Regul Sci.

[35] de Viron S, Trotta L, Steijn W (2023). Does central monitoring lead to higher quality? An analysis of key risk indicator outcomes. Ther Innov Regul Sci.

[36] de Viron S, Trotta L, Steijn W (2024). Does central statistical monitoring improve data quality? An analysis of 1,111 sites in 159 clinical trials. Ther Innov Regul Sci.

