# Supplementary material for: Central statistical monitoring in clinical trial management: A scoping review
Source: Clin Trials. Author manuscript; Available in PMC 2025 Jun 1. (PMC7617700; doi:10.1177/17407745241304059)
Supplement: Supplementary Material [file EMS205271-supplement-Supplementary_Material.zip › sj-docx-3-ctj-10.1177_17407745241304059.docx]

| **Supplemental Material 3** |
| --- |
| **Central statistical monitoring in clinical trial management: a scoping review**  Maciej Fronc, Michał Jakubczyk, Sharon B. Love, Susan Talbot, Timothy Rolfe |


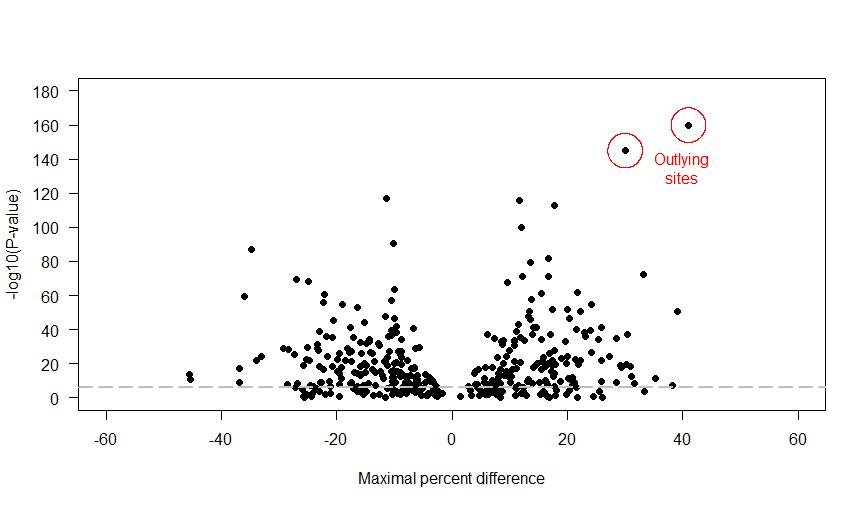


Figure S3-1. A volcano plot showing the distribution of terminal digit preference across all sites


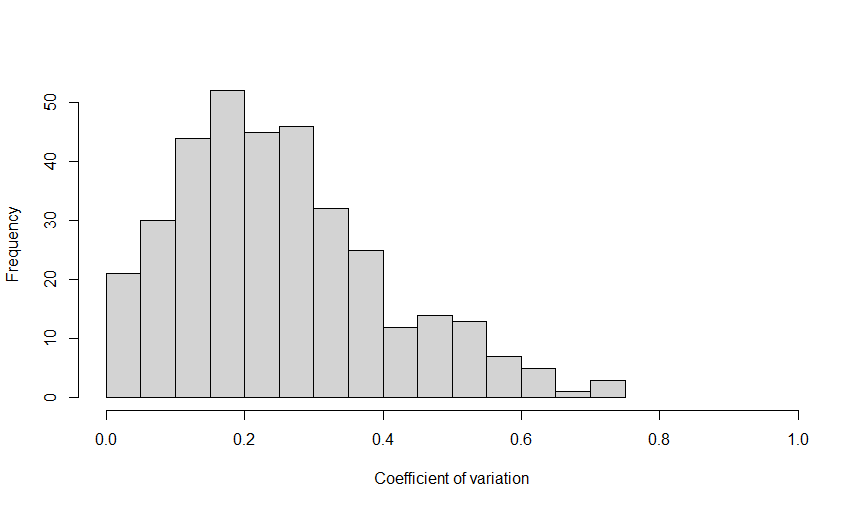


Figure S3-2. A distribution of the coefficient of variation for continuous variable

a)


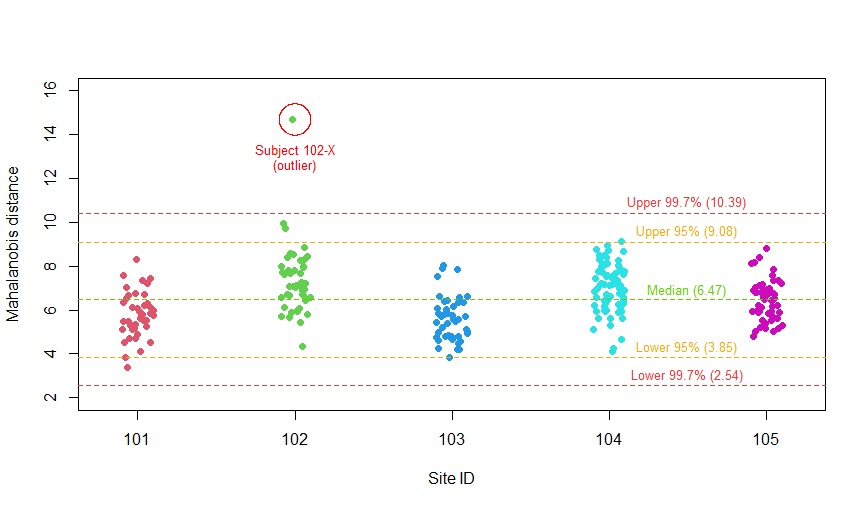


b)


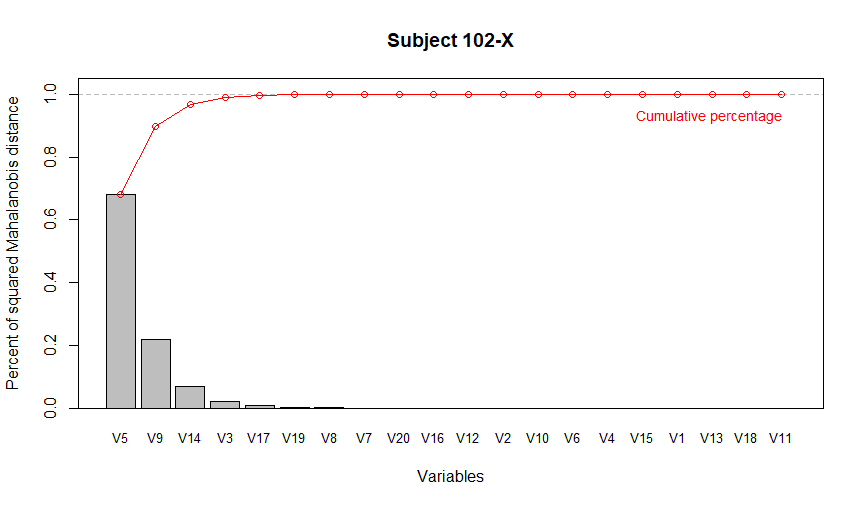


Figure S3-3. Outlier detection in terms of the Mahalanobis distance: (a) A scatterplot of Mahalanobis distances by site. Control limits (red and yellow dashed lines) are based on 95% and 99.7% Upper limits help to identify outliers, while lower limits help to identify inliers. Only one outlier (subject 102-X) was flagged. (b) A Pareto plot for patient 102-X showing the contribution of covariates to the outlier.


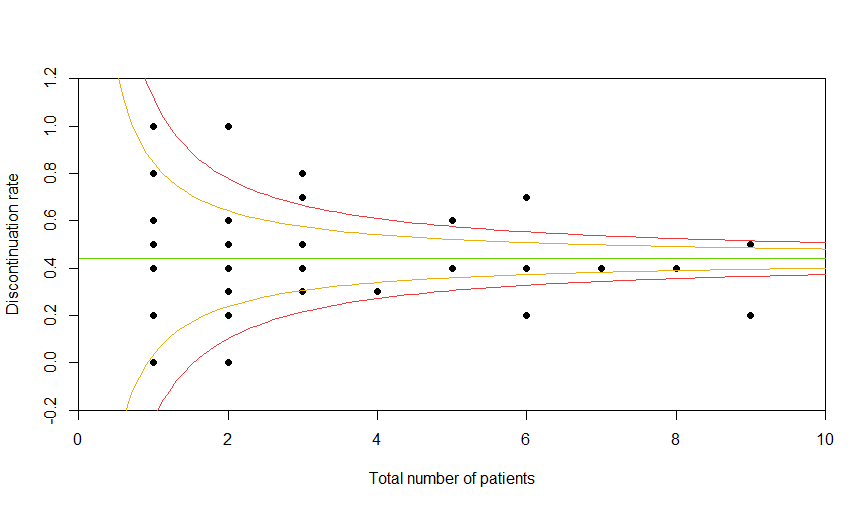


Figure S3-4. A funnel plot for the rate of discontinuation. The green line is the average discontinuation rate, while yellow and red lines are control limits based on 95% and 99.7% confidence interval.
