# Supplementary material for: Central statistical monitoring in clinical trial management: A scoping review
Source: Clin Trials. Author manuscript; Available in PMC 2025 Jun 1. (PMC7617700; doi:10.1177/17407745241304059)
Supplement: Supplementary Material [file EMS205271-supplement-Supplementary_Material.zip › sj-docx-1-ctj-10.1177_17407745241304059.docx]

| **Supplemental Material 1** |
| --- |
| **Central statistical monitoring in clinical trial management: a scoping review**  Maciej Fronc, Michał Jakubczyk, Sharon B. Love, Susan Talbot, Timothy Rolfe |

**Details on inclusion and exclusion criteria**

The papers identified in the search were subsequently analysed, and we included papers which met any of the following inclusion criteria:

- Papers addressing remote monitoring strategies of CTs that outline the theoretical framework for CSM;
- Papers presenting the currently used CSM methodology;
- Research on development of the CSM methodology;
- Papers decomposing the concept of the risk-based approach to clinical trials;
- Papers addressing the issue of misconduct in CTs in the context of quality management.

At least one reason sufficed for the article to be excluded:

- Papers with repeated subject matter. The one which contributed more to the development or explanation of CSM was selected;
- Research on on-site monitoring excluding centralized approach;
- Topics from the list of inclusion criteria are discussed only superficially;
- Papers not detailed in making data-driven decisions.
- All choices were discussed between authors at every stage of the reviewing process, and the final decision on selection of the articles was made by consensus.
