# Supplementary material for: Central statistical monitoring in clinical trial management: A scoping review
Source: Clin Trials. Author manuscript; Available in PMC 2025 Jun 1. (PMC7617700; doi:10.1177/17407745241304059)
Supplement: Supplementary Material [file EMS205271-supplement-Supplementary_Material.zip › sj-docx-2-ctj-10.1177_17407745241304059.docx]

| **Supplemental Material 2** |
| --- |
| **Central statistical monitoring in clinical trial management: a scoping review**  Maciej Fronc, Michał Jakubczyk, Sharon B. Love, Susan Talbot, Timothy Rolfe |

**CSM processes (contd.)**

Timmermans et al. distinguished four areas of CTs which contribute to the quality level of the final outcome,^1^ i.e.: study design, study conduct, analysis of the results and reporting. ICH recommends sponsors to implement a quality management system across all stages of the trial process.^2^ The system should involve measures that have an impact on quality of CTs from their early stages (Figure S2-1).


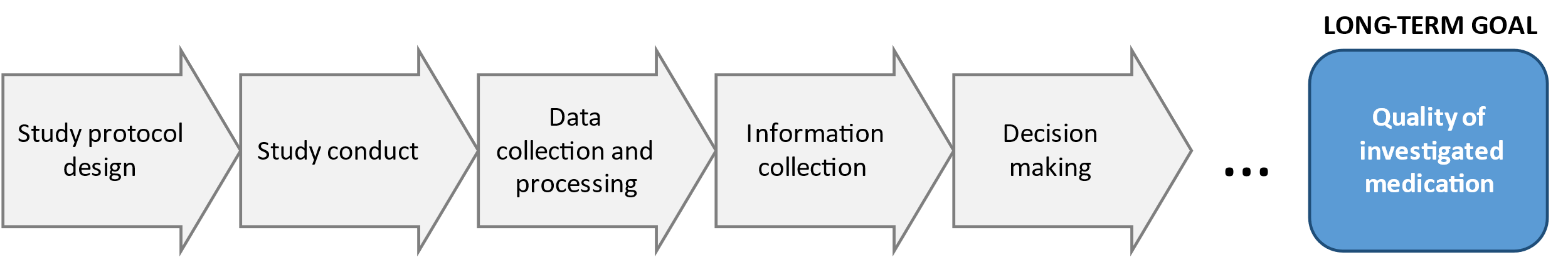


Figure S2-1. Stages of CTs impacted by quality management processes

The stage of data collection and processing is essential as it interacts indirectly with quality of the final product. Data quality is strictly dependent on data consistency which can be measured across all study sites by performing statistical analyses.^3^ Li et al. addressed the issue of data integrity – i.e. overall accuracy, completeness, and consistency of data – based on results of clinical trials published in scientific journals.^4^ They focused on statistical assessment of misconduct in CTs as a source of data integrity issues. The researchers proposed two approaches of data integrity assessment (DIA) depending on clinical data availability: DIA with individual data where all results are available, and DIA without individual data where detailed results of participants are unknown and only aggregated values are shared. The first approach utilizes methods that compare a single patient with a general tendency across the study. Even calculations are not necessary, and the whole assessment might be limited to data visualization and simple comparison of single values. In turn, the second approach based on statistical testing which provides general information about sites and determines differences between them by verifying statistical hypotheses.

The prospect of risk applied to CTs suggest to draw attention to ‘things that really matter’ within study conduct.^3^ Regulators recommend conducting risk assessment, which is particularly important, as it determines the monitoring strategy as a part of quality management. Then single subjects or sites are investigated in detail proportionate to risk distributed across the whole study. The application of RBQM aims to avoid overcharging the monitoring strategy and improve its efficiency.^5^ Monitoring conducted respectively to study risk is called risk-based monitoring (RBM) and it is a component of the RBQM concept. RBM capitalises on monitoring conducted in the centralized way based on remote evaluation of study conduct and data capture.^1^ This approach supports operationalization of the RBM concept.^3^ What is more, Timmermans et al. recognized RBM as a new paradigm of quality control.^6^ Effects of RBM guides researchers in applying reasonable actions seeking to deliver an expected quality level of a final product. Moreover, Hatayama and Yasui appeal to Pareto principle (otherwise known as the 80:20 rule)^7^ as pivotal concept of quality management,^8^ which is consistent with the CSM concept. While intuition may suggest that irregularities are limited to a minority of study sites, this minority often contributes to the majority of errors in study conduct. This point of view connects CSM with quality management within an indirect relation (Figure S2-2).


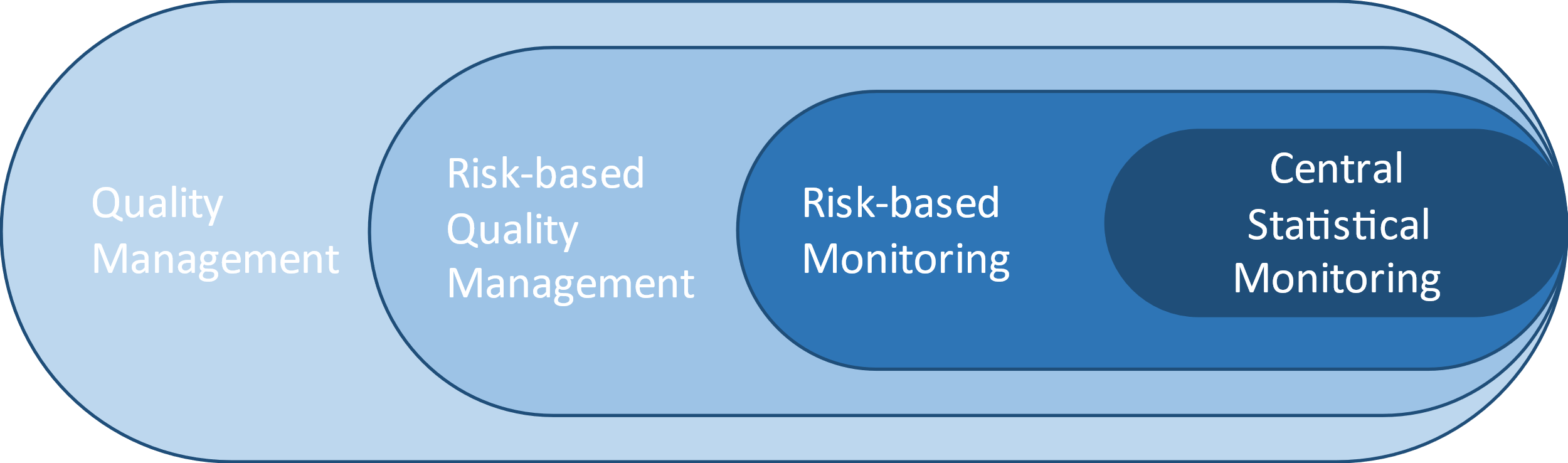


Figure S2-2. Linkage of the concepts underlying the centralised monitoring

*Where CSM is located in the CT workflow and how it operates?*

The goal of a clinical trial protocol is to provide a suitably homogeneous population in which to reliably characterise the effects of the intervention under investigation. As such, there should not be any major data discrepancies as all sites follow the same procedures within the single study. Any discrepancies could be minor but ultimately may increase the risk of study failure. Buyse et al. indicated typical causes of data issues such as fraud, data tampering, sloppiness, or miscalibration.^3^ All of them might jeopardise the study outcome. Although it is difficult to capture them with naked eye, these causes leave signals detectable by applied algorithms. The signals are usually related to missing values or wrong number of records (reporting), atypical distribution of a feature (data tendency), observable change of a parameter over time (visit-to-visit evolution), or atypically reported visit days (visit date).^6^ Signals and their causes are closely linked making them interdependent (Figure S2-3).


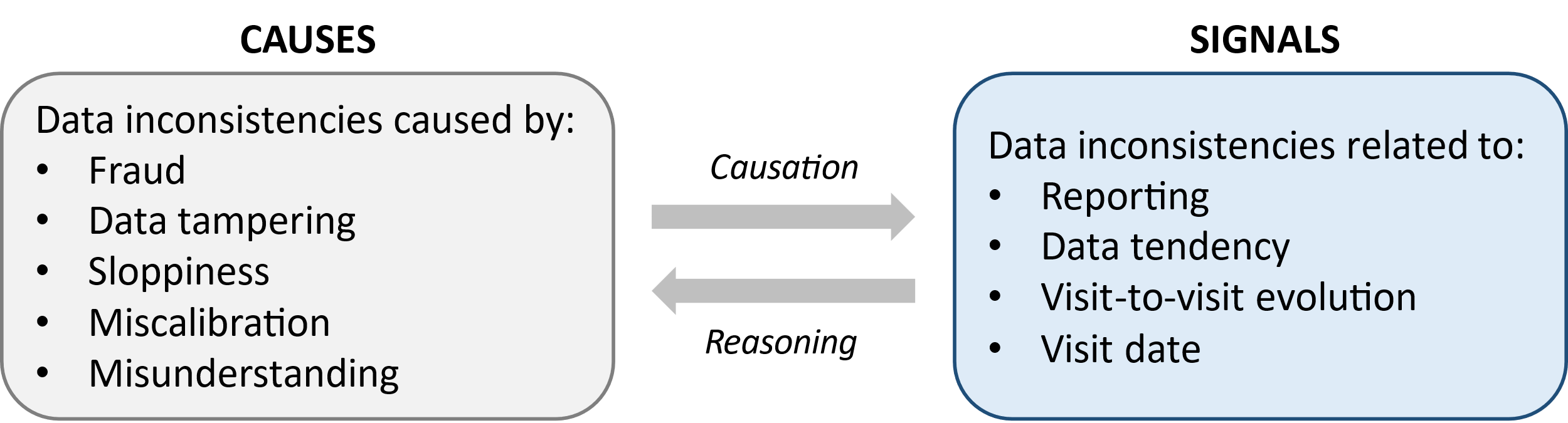


Figure S2-3. Interdependence of causes and signals

Sloppiness and miscalibration happen unintentionally, however, fraud and data tampering are committed consciously. Cragg et al. noticed that two thirds of articles about RBM they reviewed concerned CSM and a half of them addressed detection of fabricated data in CTs.^5^ It shows a popularity of this methodology resulting from the scale of fraudulent activity.

Fraud easily evades human cognition but is detectable by using statistical approach.^9^ Humans are unable to come up with credible data due to their incomplete knowledge of how real data are generated.^4^ Herson defined two strategies for dealing with fraud – defensive and offensive ones.^10^ Defensive strategy works in the spirit of quality by design philosophy, and it aims to prevent fraud by proper study planning. In turn, offensive strategy involves reacting to fraud detected through CSM, and implementing mitigation of the risk (Figure S2-4).


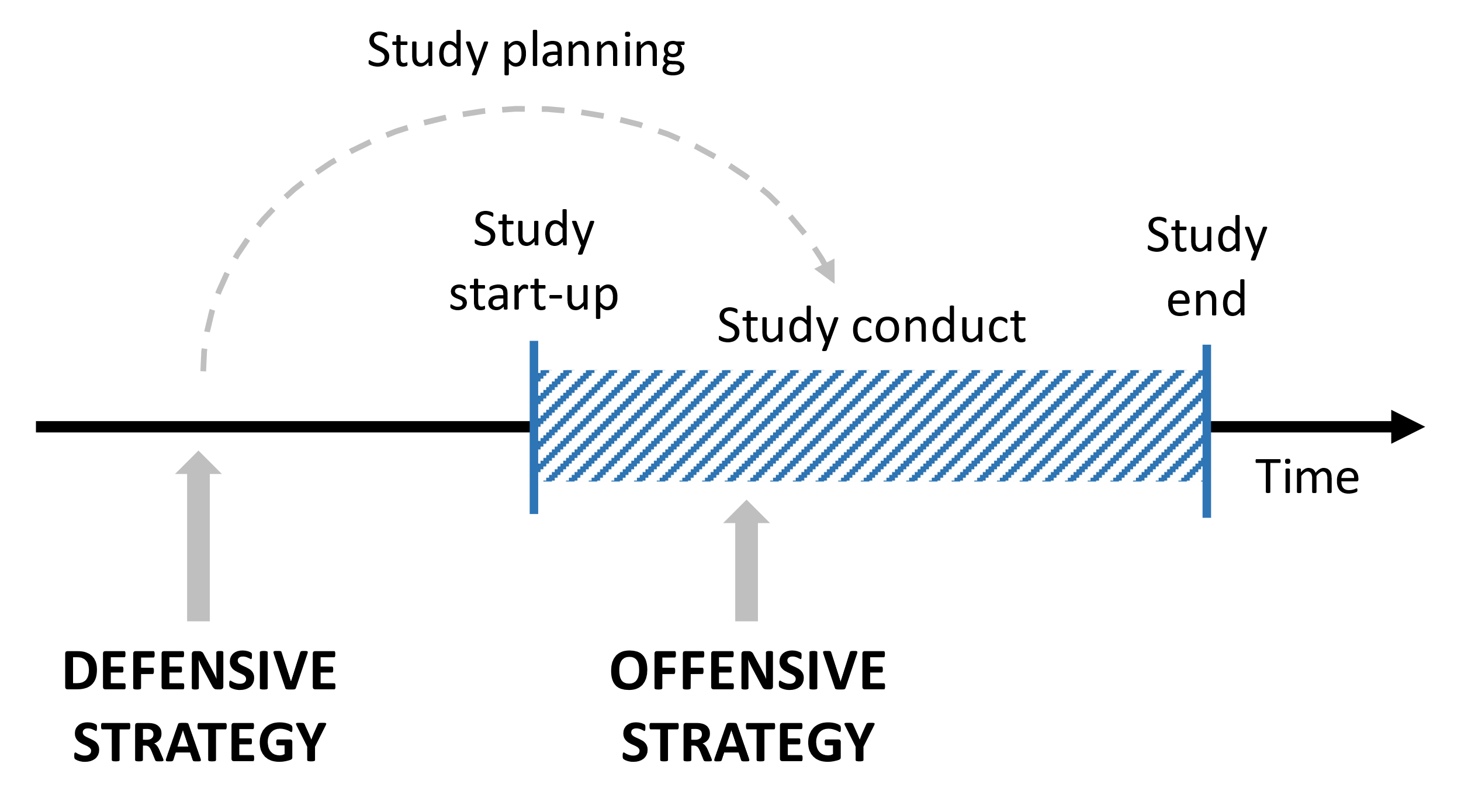


Figure S2-4. Timeline of defensive and offensive strategies application

Application of CSM decreases risk across the study. Identified KRIs require further investigation and application of preventive measures. KRIs are specific to the study, therefore they need to be customized to conduct targeted monitoring. Timmermans et al. even recommends not to have any prior assumption before statistical analysis to prevent omitting any risks.^6^ Signals detected by using statistical tools are a premise for targeted source data review (SDR) and if warranted, source data verification (SDV) at sites marked with ‘red flags’. CSM facilitates decision-making on the necessity to conduct SDV/SDR.^5^ The outcome of CSM helps to avoid a need for full SDV/SDR at all sites across the study (Figure S2-5).


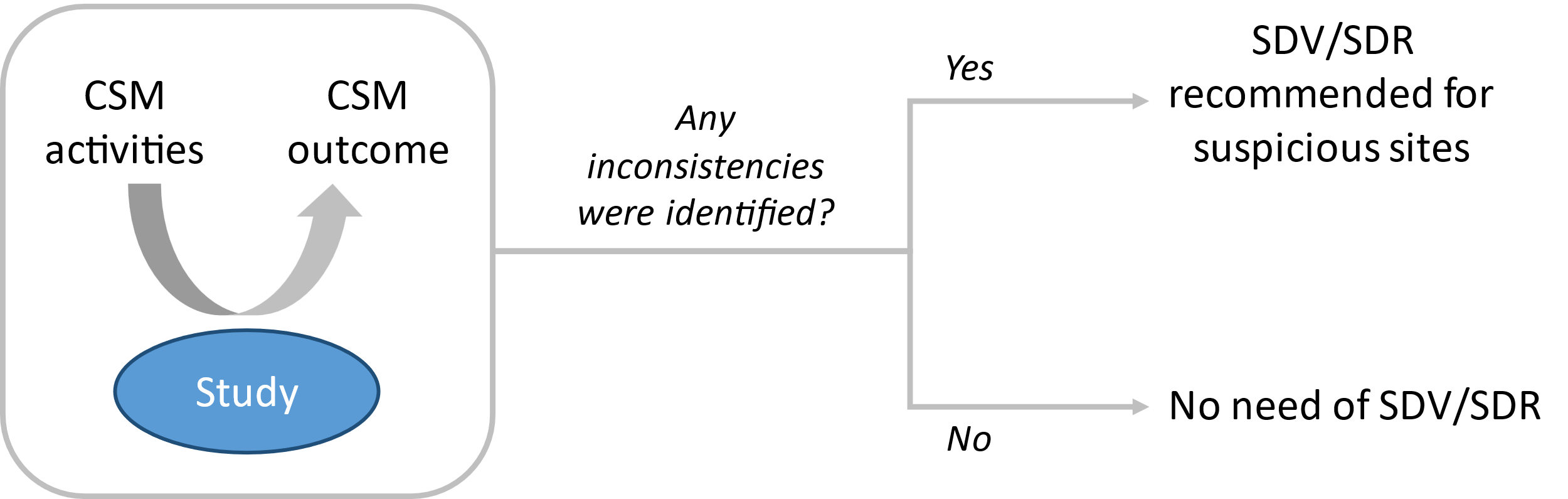


Figure S2-5. Decision-making involving the CSM outcome

CSM is a likely cheaper alternative to routine on-site monitoring of all sites within a study. Apart from the advantages of CSM, there are also some limitations. The currently used methods are selective and underperform in the case of small trials.^5,11^ What is more, developed algorithms are often tested on fabricated data instead of using real-world data. Data are fabricated for this purpose with certain assumptions which might turn out to be incorrect and not reflect the inner nature of clinical data. Then, the algorithm may underperform and give unsatisfactory results. Kirkwood et al. encourage other researchers to refine these algorithms on their own datasets, and especially from actual CTs.^11^

**References**

1. Timmermans C, Venet D, Burzykowski T. Data-driven risk identification in phase III clinical trials using central statistical monitoring. *Int J Clin Oncol* 2016; 21: 38–45.

2. International Council for Harmonisation of Technical Requirements for Pharmaceuticals for Human Use (ICH). *Guideline Good Clinical Practice E6(R2)*, https://www.ema.europa.eu/en/documents/scientific-guideline/ich-e-6-r2-guideline-good-clinical-practice-step-5_en.pdf (2016).

3. Buyse M, Trotta L, Saad ED, et al. Central statistical monitoring of investigator-led clinical trials in oncology. *Int J Clin Oncol* 2020; 25: 1207–1214.

4. Li W, Bordewijk EM, Mol BW. Assessing research misconduct in randomized controlled trials. *Obstet Gynecol* 2021; 138: 338–347.

5. Cragg WJ, Hurley C, Yorke-Edwards V, et al. Dynamic methods for ongoing assessment of site-level risk in risk-based monitoring of clinical trials: A scoping review. *Clin Trials* 2021; 18: 245–259.

6. Timmermans C, Doffagne E, Venet D, et al. Statistical monitoring of data quality and consistency in the Stomach Cancer Adjuvant Multi-institutional Trial Group Trial. *Gastric Cancer* 2016; 19: 24–30.

7. Hatayama T, Yasui S. Bayesian central statistical monitoring using finite mixture models in multicenter clinical trials. *Contemp Clin Trials Commun* 2020; 19: 100566.

8. Juran J, Godfrey A. *Juran’s quality handbook*. wyd. 5. New York: McGraw-Hill Professional Publishing, 1998.

9. Fronc M, Jakubczyk M. From business to clinical trials: a systematic review of the literature on fraud detection methods to be used in central statistical monitoring. *Przegląd Stat* 2022; 69: 1–31.

10. Herson J. Strategies for dealing with fraud in clinical trials. *Int J Clin Oncol* 2016; 21: 22–27.

11. Kirkwood AA, Cox T, Hackshaw A. Application of methods for central statistical monitoring in clinical trials. *Clin Trials* 2013; 10: 783–806.
